# Impact of Artificial Intelligence‐Assisted Endoscopy on Screening for Upper Gastrointestinal Cancer in a Large‐Scale Health Screening Facility

**DOI:** 10.1111/den.70159

**Published:** 2026-05-01

**Authors:** Chihiro Goto, Ryo Nakagawa, Ryosuke Horio, Akane Kurosugi, Tatsuya Kaneko, Tsubasa Ishikawa, Yuki Ohta, Takashi Taida, Kenichiro Okimoto, Tomoaki Matsumura, Jun Kato

**Affiliations:** ^1^ Department of Gastroenterology Chiba University Chiba Japan; ^2^ Omiya City Clinic Saitama Japan

**Keywords:** artificial intelligence, cancer screening, esophageal cancer, gastric cancer, propensity score

## Abstract

**Objectives:**

Artificial intelligence (AI)‐assisted endoscopy has been developed for the early detection of upper gastrointestinal cancer; however, its clinical effectiveness remains insufficiently evaluated. This study assessed its effectiveness in a health screening facility.

**Methods:**

This retrospective cohort study compared AI‐assisted and non‐AI‐assisted upper gastrointestinal endoscopy at Omiya City Clinic, Japan (April 2021–March 2024). Participants who underwent endoscopy between April 2021 and March 2023 were classified as the non‐AI group, while those examined between April 2023 and March 2024 comprised the AI group. The AI‐assisted system was introduced in April 2023. The primary outcome was cancer detection rate (CDR), with secondary outcomes including biopsy rate and positive predictive value (PPV). Propensity score matching (PSM) was performed for age, sex, alcohol consumption, smoking, *Helicobacter pylori* infection history, endoscopist experience, and prior‐year endoscopy to minimize bias.

**Results:**

In total, 17,662 were included in the AI group and 32,318 in the non‐AI group. PSM created 17,662 matched pairs. In the AI group, the CDR for gastric cancer (GC) was significantly higher compared to the non‐AI group (0.10% vs. 0.03%, *p* < 0.05). The biopsy rate was slightly higher in the AI group, with no significant difference, whereas the PPV of biopsy for gastric cancer and esophageal cancer was significantly increased (4.84% vs. 2.16%, *p* < 0.05).

**Conclusions:**

In a clinical screening setting, AI‐assisted endoscopy significantly improved the CDR of GC and enhanced the PPV of biopsies. These findings highlight AI‐assisted endoscopy as a valuable tool for early GC diagnosis in screening environments.

## 1 Introduction

In recent years, early endoscopic treatment for upper gastrointestinal (UGI) cancers, such as esophageal and gastric cancers (GC), has increased in Japan and globally [1]. Compared to traditional surgical treatment, endoscopic treatment offers significant quality‐of‐life improvements and the potential for curative outcomes provided that appropriate lesions are detected and resected [2, 3]. In high *H. pylori* prevalence regions like Japan and South Korea, GC incidence remains high [4, 5], and voluntary health checkups are common for early detection [6].

However, early detection remains challenging due to limited examination time, varying endoscopist experience, tumor location, or posteradication status [7, 8, 9, 10]. The British Society of Gastroenterology and the Association of UGI Surgeons define “missed UGI cancers” as those cases where a patient was diagnosed within 3 years of a prior endoscopy [11]. It was observed that 6.0%–11.3% of UGI cancers are missed [12, 13]. In response to diagnostic challenges, recent research has investigated the application of artificial intelligence (AI) to improve early detection of UGI cancers [14]. The primary function of endoscopic AI, computer‐aided detection (CADe), automatically detects and localizes suspicious areas and identifies key anatomical landmarks. These features guide endoscopists during examination, helping to reduce missed diagnoses and improve diagnostic accuracy [15]. Many studies have assessed the diagnostic performance of endoscopic AI using prerecorded images or videos [16, 17, 18]; however, evidence of its effectiveness in real clinical settings remains limited. In China, several prospective studies have been conducted [19]; focusing mainly on evaluating AI's sensitivity and specificity rather than determining whether its use increases actual CDR. In gastrointestinal endoscopy, AI has been widely adopted for colonoscopy, where the target—colorectal adenoma—is common and easily evaluated through adenoma detection rates [20]. In contrast, due to the low incidence rates of gastric and esophageal cancers (EC), real‐world clinical data on AI‐assisted upper gastrointestinal endoscopy remains scarce, highlighting the importance of reporting clinical outcomes in this field [21].

We used data from a large‐scale voluntary health screening facility to evaluate the impact of artificial intelligence–assisted upper gastrointestinal endoscopy on the detection of gastric and esophageal cancers in real‐world clinical practice.

## 2 Methods

### 2.1 Participants

The study included individuals who underwent UGI endoscopy as part of a health screening at the Omiya City Clinic (Saitama City) between April 2021 and March 2024. Because a large proportion of individuals underwent repeated annual EGDs as part of routine health checkups, examinations were analyzed on a per‐year basis, and repeated examinations in different years were included as separate observations. At the initial visit, all participants undergoing the health screening underwent a *H. pylori* antibody test, and their history of infection and eradication was assessed through a standardized questionnaire. An AI‐assisted system was introduced in April 2023, and all endoscopic examinations were conducted using this device after that. Participants between April 2021 and March 2023 were classified into the non‐AI group, whereas those between April 2023 and March 2024 were categorized into the AI group. Regarding cancer diagnosis, cases were counted as cancer if they were diagnosed as cancer on biopsy at the health screening facility or pathologically confirmed after referral to a specialized institution. Gastric cancer was defined as histopathologically confirmed gastric adenocarcinoma, diagnosed on biopsy or resected specimens, according to the World Health Organization (WHO) classification. Both early and advanced GCs were included. Adenomas and nonepithelial tumors were excluded. Foveolar‐type gastric tumors [22] were classified as adenomas according to the WHO classification. Esophageal cancer was defined as histopathologically confirmed malignant epithelial tumors of the esophagus, including esophageal squamous cell carcinoma and esophageal adenocarcinoma, based on the WHO classification. No individuals were diagnosed with cancer more than once during the study period; therefore, repeated cancer diagnoses in the same individual did not affect the analysis.

### 2.2 Endoscopic Procedure

**FIGURE 1:**
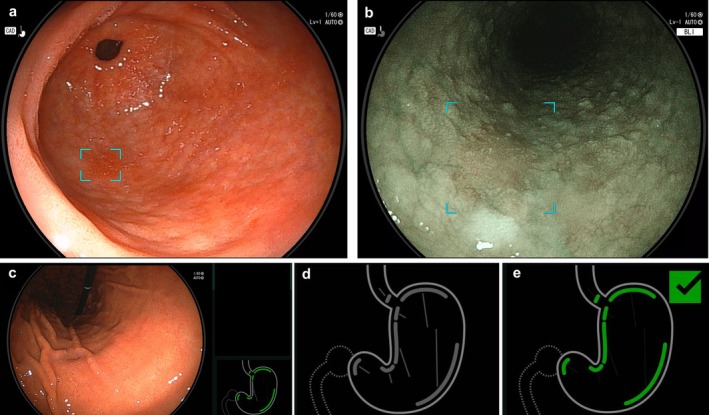
Functions of the artificial intelligence instruments used in the study. (a) Detection of areas with potential gastric tumor lesions during white light imaging observation. (b) Detection of areas with potential esophageal squamous cell carcinoma during blue laser imaging observation. (c) The actual display of the landmark photo checker during endoscopic procedure. (d) Initial state of the landmark photo checker. (e) Complete state of the landmark photo checker.

Endoscopic examinations were performed using five light‐source systems. One of the five systems utilized a light source (VP‐4450/XL4450) and UGI endoscopes (EG‐580NW and EG‐580NW2) (FUJIFILM Co., Tokyo, Japan) between April 2021 and March 2023. The other four systems employed a LASEREO system (LL‐7000/VP‐7000) (FUJIFILM Co., Tokyo, Japan) and UGI endoscopes (EG‐L580NW7) (FUJIFILM Co., Tokyo, Japan). Examinations were performed almost evenly across the five light‐source systems. 20Starting in April 2023, all five systems were standardized using the LASEREO system (LL‐7000/VP‐7000), UGI endoscopes (EG‐L580NW7), and an AI‐assisted endoscopy system (EW10‐EG01) (FUJIFILM Co., Tokyo, Japan). EW10‐EG01 features a CADe mode that highlights suspected lesions of esophageal squamous cell carcinoma (ESCC) and gastric tumors with blue boxes (Figure 1). According to the package insert [23], the sensitivity for detecting suspected squamous cell carcinoma is 98.6% with blue laser imaging (BLI) and 97.6% with linked color imaging (LCI); specificity is 94.2% and 97.0%, respectively. For detecting gastric tumor–like lesions, sensitivity is 95.5% with white light imaging (WLI) and 94.6% with LCI, whereas specificity is 95.7% and 98.0%, respectively. The device also includes a “Landmark Photo Checker” that confirms sufficient observation of key gastric regions (Figure 1). The EW10‐EG01 system used with version 1.

All examinations were performed without sedation. During observation, various image‐enhanced endoscopy techniques [24], such as BLI, LCI, Fujinon Intelligent Color Enhancement, and chromoendoscopy with indigo carmine dye [25], were used at the discretion of the endoscopist in addition to WLI. Even when lesions were flagged by the AI system, the final decision to perform a biopsy was made by an endoscopist. During the study period, there were 91 endoscopists with confirmed experience levels who participated, while several others lacked recorded experience due to 1‐day shifts. Examination time was measured from patient registration in the endoscopic system to documentation of findings and recorded on a minute basis. Clearly erroneous data, such as cases where the end time preceded the start time, were excluded from the analysis.

### 2.3 Clinical Outcomes

The primary outcome was CDR, defined as the proportion of individuals diagnosed with histopathologically confirmed EC or GC during each endoscopic examination. Adenomas and dysplastic lesions without histological evidence of invasive carcinoma were not classified as cancer.

Secondary outcomes included the biopsy rate, defined as the proportion of examinations in which at least one biopsy was performed, and the positive predictive value (PPV) of biopsy for cancer, defined as the number of individuals diagnosed with GC or EC divided by the total number of individuals who underwent biopsy. When multiple biopsies were obtained from the same individual during a single examination, the pathological diagnosis was determined on a per‐patient basis, and each individual was counted once in the PPV analysis.

As a subanalysis, examination times before and after the introduction of AI were evaluated, and the clinicopathological characteristics of GC detected were analyzed.

### 2.4 Propensity Score Matching (PSM)

PSM was performed to reduce selection bias in the non‐AI and AI groups using a greedy nearest‐neighbor 1:1 matching protocol without replacement and a caliper width equivalent to 0.2 of the standard deviation for the logit of the propensity score [26]. Alcohol consumption and smoking are universal risk factors for ESCC and GC [27, 28]. *H. pylori* infection is a well‐established risk factor for GC [5]. Both EC and GC have higher incidence rates in men and older patients. Factors potentially influencing CDR include the skill level of the endoscopist and the reduced likelihood of identifying new lesions if an endoscopy was performed in the previous year. To account for these factors, logistic regression was used to calculate propensity scores based on the following variables: age, sex, smoking status (ever vs. never), and alcohol use (current drinker vs. non‐drinker), history of *H. pylori* infection (present vs. absent), endoscopist experience, and whether endoscopy had been performed in the previous year (yes vs. no). Endoscopist experience was categorized into three groups based on years of practice (5–9, 10–14, and ≥ 15 years). Clinical outcomes were compared between the two matched groups. Smoking status was classified as ever or never smoker. Alcohol consumption was categorized as current drinker or nondrinker, with nondrinker including never and former drinkers. *H. pylori* infection status was dichotomized as present or absent, with “present” including participants with current *H. pylori* infection or a history of eradication therapy and “absent” including those without evidence of *H. pylori* infection, based on medical history and/or test results obtained at the time of health checkup.

### 2.5 Statistical Analysis

The baseline characteristics and outcomes were analyzed using the chi‐squared test for categorical data and the Mann–Whitney U test for continuous data. Comparisons were made between the AI and non‐AI groups regarding CDR, biopsy rates, and the PPV of biopsies. For the subanalysis, data before matching were used due to the limited number of cases. All analyses were conducted using complete cases only, with any cases containing missing data excluded. Statistical significance was set at *p* < 0.05 for all tests. Statistical analyses were performed using JMP version 18.0.0 (SAS Institute Inc., Cary, NC, USA).

## 3 Results

### 3.1 Baseline Characteristics Before PSM

**FIGURE 2:**
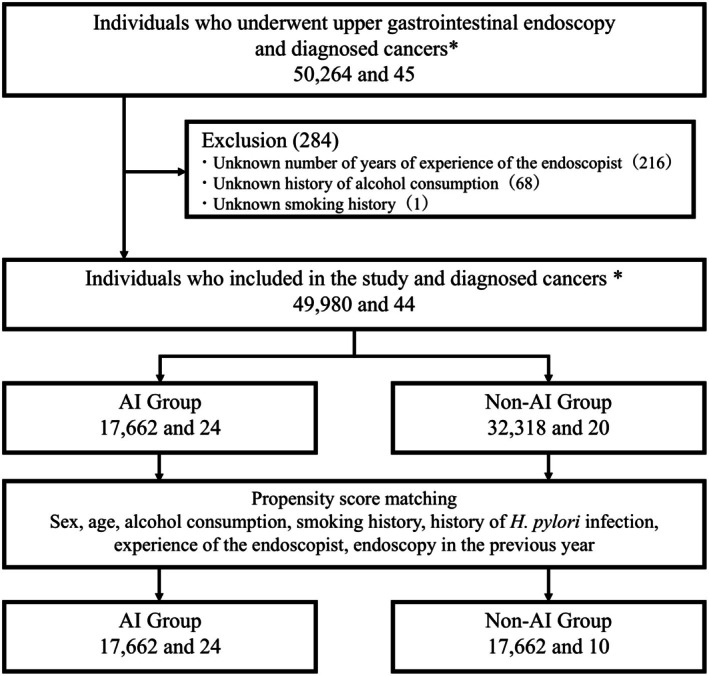
Flowchart of the study participants, cancer diagnoses, and matching process. *The number of participants diagnosed with gastric cancer or esophageal carcinoma.

Between April 2021 and March 2024, 50,264 individuals underwent UGI endoscopy as part of voluntary screening. Participants lacking valid questionnaire responses on drinking or smoking history and those for whom endoscopist experience could not be confirmed due to spot work were excluded. As shown in Figure 2, 49,980 individuals were analyzed.

The AI and non‐AI groups comprised 17,662 and 32,318 individuals, respectively. Before matching, 44 individuals were diagnosed with cancer: 31 GC (18 AI, 13 non‐AI) and 14 EC (6 AI, 8 non‐AI). One non‐AI case had both GC and EC. Table 1 shows baseline characteristics. The non‐AI group had significantly more participants with prior *H. pylori* infection, whereas the AI group had more who had undergone endoscopy in the previous year. Endoscopist experience also differed significantly, but other variables did not. Among 91 endoscopists with known experience, the range was 5–37 years (median 11).

**TABLE 1:** Characteristics of examinees before PSM.

	AI group (*n* = 17,662)		Non‐AI group (*n* = 32,318)		*p*
Sex, male, %	10,660	60.35%	19,406	59.73%	0.1746
Age, mean, ±SD	52.03	10.23	51.89	10.21	0.0880
History of *H. pylori* infection^a^					0.0014
Present, %	4446	25.17%	8557	26.48%	
Absent, %	13,218	74.83%	23,762	73.52%	
Prior‐year endoscopy					< 0.0001
Present, %	11,202	63.42%	19,160	59.28%	
Absent, %	6462	36.58%	13,159	40.72%	
Smoking status					0.1344
Ever, %	7760	43.93%	14,423	44.63%	
Never, %	9904	56.07%	17,896	55.37%	
Alcohol consumption					0.0734
Current drinker, %	11,155	63.15%	20,148	62.34%	
Nondrinker^b^, %	6509	36.85%	12,171	37.66%	
Experience of the endoscopist					< 0.0001
5–9	7645	43.29%	11,792	36.49%	
10–14	6622	37.49%	12,441	38.50%	
≥ 15	3395	19.22%	8085	25.02%	

Abbreviation: SD, standard deviation.

^a^ *H. pylori* infection was defined as present (current infection or history of eradication) or absent (never infected).

^b^ Nondrinker including never and former drinkers.

### 3.2 Matched Factors After PSM

PSM generated 17,662 matched pairs (Figure 2). Post‐matching comparisons (Table 2) showed no significant differences in any factors between groups. All absolute standardized differences were within acceptable limits, confirming balance after PSM.

**TABLE 2:** Characteristics of examinees after PSM.

	AI group (*n* = 17,662)		Non‐AI group (*n* = 17,662)		*p*	ASD
Sex, male, %	10,659	60.35%	10,734	60.77%	0.4142	0.009
Age, mean, ±SD	52.03	9.94	52.03	10.23	0.8836	0.001
History of *H. pylori* infection^a^					0.8733	0.002
Present, %	4445	25.17%	4432	25.09%		
Absent, %	13,217	74.83%	13,230	74.91%		
Prior‐year endoscopy					0.8510	0.002
Present, %	11,201	63.42%	11,218	63.51%		
Absent, %	6461	36.58%	6444	36.49%		
Smoking status					0.6218	0.005
Ever, %	7759	43.93%	7713	43.67%		
Never, %	9903	56.07%	9949	56.33%		
Alcohol consumption					0.4330	0.008
Current drinker, %	11,154	63.15%	11,225	63.55%		
Nondrinker^b^, %	6508	36.85%	6437	36.45%		
Experience of the endoscopist					0.9116	0.005
5–9	7645	43.29%	7605	43.06%		
10–14	6622	37.49%	6647	37.63%		
≥ 15	3395	19.22%	3410	19.31%		

Abbreviations: ASD, absolute standardized differences; SD, standard deviation.

^a^ *H. pylori* infection was defined as present (current infection or history of eradication) or absent (never infected).

^b^ Nondrinker including never and former drinkers.

### 3.3 Comparison of CDR

After PSM, 24 individuals were diagnosed with GC (18 AI [0.10%], 6 non‐AI [0.03%]), indicating a significantly higher rate in the AI group. Ten individuals were diagnosed with EC (6 AI [0.03%], 4 non‐AI [0.02%]), showing a nonsignificant increase. Overall, 34 individuals had either GC or EC (24 AI [0.14%], 10 non‐AI [0.06%]), again significantly higher in the AI group (Table 3).

**TABLE 3:** The CDR after PSM.

	AI group (*n* = 17,662)		Non‐AI group (*n* = 17,662)		*p*
GC, CDR (%)	18	0.10%	6	0.03%	0.0122
EC, CDR (%)	6	0.03%	4	0.02%	0.5256
GC + EC, CDR (%)	24	0.14%	10	0.06%	0.0148

Abbreviations: CDR, cancer detection rate; EC, esophageal carcinoma; GC, gastric cancer; PSM, propensity score matching.

In a supplementary analysis stratified by endoscopist experience (Figure S1), a significant increase in cancer detection rates was observed in the AI group among less experienced endoscopists (5–9 years), whereas no significant differences were observed in more experienced groups (Table S1).

### 3.4 Comparison of Biopsy Rates and PPV

Table 4 shows biopsy rates and PPV after PSM. The gastric biopsy rate was 2.28% in the AI group and 2.12% in the non‐AI group, with no statistically significant difference. The esophageal biopsy rate was 0.57% versus 0.53%, not significant. Combined, the biopsy rate was 2.81% in the AI group and 2.62% in the non‐AI group (*p* < 0.05).

**TABLE 4:** Comparison of biopsy rates and PPV after PSM.

Biopsy	AI group (*n* = 17,662)		Non‐AI group (*n* = 17,662)		*p*
For GC, %	403	2.28%	374	2.12%	0.2927
For EC, %	100	0.57%	93	0.53%	0.6133
For GC + EC, %	496	2.81%	463	2.62%	0.2799
	**Biopsy for GC AI group (*n* = 403)**		**Biopsy for GC Non‐AI group (*n* = 374)**		***p***
GC diagnosis, PPV (%)	18	4.47%	6	1.60%	0.0182
	**Biopsy for EC AI group (*n* = 100)**		**Biopsy for EC Non‐AI group (*n* = 93)**		***p***
EC diagnosis, PPV (%)	6	6.00%	4	4.30%	0.5932
	**Biopsy for GC + EC AI group (*n* = 496)**		**Biopsy for GC + EC Non‐AI group (*n* = 463)**		***p***
GC + EC diagnosis, PPV (%)	24	4.84%	10	2.16%	0.0227

Abbreviations: EC, esophageal cancer (including both esophageal squamous cell carcinoma and adenocarcinoma); GC, gastric cancer; PPV, positive predictive value; PSM, propensity score matching.

The PPV of gastric biopsies was 4.47% in the AI group and 1.60% in the non‐AI group, and that of esophageal biopsies was 6.00% and 4.30%, respectively. The combined PPV of gastric and esophageal biopsies was 4.84% in the AI group and 2.16% in the non‐AI group. The PPV of gastric biopsy and that of combined esophageal and gastric biopsy were both significantly higher in the AI group.

### 3.5 Comparison of Examination Time

To ensure accuracy, records with end times earlier than start times (*n* = 345) and extreme outliers based on interquartile range (*n* = 11) were excluded. The average examination time was 7.22 ± 2.15 min in the AI group and 7.37 ± 2.20 min in the non‐AI group. Although the difference was small, the examination time was significantly shorter in the AI group (*p* < 0.0001; Table 5).

**TABLE 5:** Comparison of examination time after PSM.

	AI group (*n* = 17,508)		Non‐AI group (*n* = 17,549)		*p*
Examination time, mean, ±SD	7.22	2.15	7.37	2.20	< 0.0001

*Note:* To ensure the accuracy of the analysis, clearly incorrect records—such as those in which the end time was earlier than the start time—were excluded (*n* = 345). In addition, outliers were defined based on the interquartile range of the examination time distribution, and extreme values were also excluded (*n* = 11).

### 3.6 Comparison in GC Cases

Because of the limited number of cases, Table 6 presents an analysis of GC cases using prematching data. A total of 31 cases were included in the analysis. There were no significant differences between the groups in baseline characteristics. Histological findings were categorized into differentiated type, undifferentiated type, and other types.

**TABLE 6:** Comparison of cases diagnosed with gastric cancer before PSM.

	AI group (*n* = 18)		Non‐AI group (*n* = 13)		*p*
Sex, male, %	16	88.89%	11	84.62%	0.7276
Age, mean, ±SD	63.67	9.32	68.61	7.02	0.0957
History of *H. pylori* infection^a^					0.6573
Present, %	15	83.33%	10	76.92%	
Absent, %	3	16.67%	3	23.08%	
Endoscopy in the previous year					0.4379
Present, %	10	55.56%	9	69.23%	
Absent, %	8	44.44%	4	30.77%	
Extent of atrophy					0.4761
Closed type	6	33.33%	2	15.38%	
Open type	9	50.00%	9	69.23%	
No atrophy	3	16.67%	2	15.38%	
Smoking status					0.1411
Ever, %	13	72.22%	6	46.15%	
Never, %	5	27.78%	7	53.85%	
Alcohol consumption					0.7387
Current drinker, %	10	55.56%	8	61.54%	
Nondrinker^b^, %	8	44.44%	5	38.46%	
Experience of the endoscopist					0.3795
5–9	10	55.56%	4	30.77%	
10–14	5	27.78%	6	46.15%	
≥ 15	3	16.67%	3	23.08%	
Tumor size (mm), mean, ±SD	13.00	7.87	19.23	7.53	0.0179
Tumor size					0.0082
> 10 mm, %	9	50.00%	12	92.31%	
≤ 10 mm, %	9	50.00%	1	7.69%	
Histological type					0.5363
Differentiated, %	14	77.78%	10	76.92%	
Undifferentiated, %	3	16.67%	1	7.69%	
Others types^c^, %	1	5.56	2	15.38	
Final treatment method					0.1726
Endoscopic, %	15	83.33%	8	61.54%	
Surgery or chemotherapy, %	3	16.67%	5	38.46%	

^a^ *H. pylori* infection was defined as present (current infection or history of eradication) or absent (never infected).

^b^ Nondrinker including never and former drinkers.

^c^ The “Other types” category comprised gastric adenocarcinoma of fundic‐gland type and cases described as carcinoma in referral letters for which the histological subtype was unavailable.

Poorly differentiated adenocarcinoma (por) and signet‐ring cell carcinoma (sig) were classified as the undifferentiated type, whereas gastric adenocarcinoma of fundic gland type [29] and cases with unknown detailed histological subtype were classified as others. No significant differences were observed in the histological findings.

However, GCs detected in the AI group were significantly smaller in size. Notably, the number of small lesions measuring ≤ 10 mm was significantly higher in the AI group.

## 4 Discussion

To the best of our knowledge, this study is the first study to demonstrate that AI‐assisted endoscopy improves the detection rate of GC in real‐world clinical practice. AI‐assisted endoscopy increased GC detection, particularly of small lesions (≤ 10 mm), suggesting earlier diagnosis. Although gastric biopsies increased, biopsy PPV also improved, contributing to higher diagnostic yield. Examination time was not prolonged; rather, it was modestly shortened. AI improves both the detection rate of GC and the overall detection rate during upper gastrointestinal endoscopy at health screening facilities.

A supplementary analysis suggested that the potential benefit of AI assistance may be more pronounced among less experienced endoscopists. However, the number of cancer cases was limited, particularly in the most experienced group, and these findings should be interpreted cautiously as exploratory.

Although AI‐assisted UGI endoscopy is increasingly adopted, its real‐world effectiveness remains uncertain. A major challenge in evaluating clinical impact is the low prevalence of GC and EC. In East Asia, *H. pylori* infection contributes to high GC incidence, yet even here, accumulating adequate cases for prospective validation is time‐consuming. Reported endoscopic GC detection rates in Japanese screening facilities range from 0.30% to 0.87% [30]. In this large‐scale analysis of 49,980 individuals (7.5% of Saitama City's working‐age population) [31], AI significantly improved GC detection. The large sample size from a single high‐volume center strengthens the generalizability and supports AI integration into population‐based screening.

No significant increase in EC detection was observed, likely due to its lower prevalence and limited case numbers [32]. Accordingly, the present results regarding EC should be interpreted cautiously due to the limited statistical power. Extending the observation period could accumulate more EC cases, enabling further evaluation of AI's diagnostic benefit.

Previous AI studies [19, 33] have identified normal structures or gastritis with erythema, atrophy, or intestinal metaplasia as common false positives. Similarly, the EW10‐EG01 system sometimes reacts to normal folds or inflammatory changes. Although skilled endoscopists can distinguish normal anatomy, differentiating inflammation from early cancer may require biopsy. In this study, biopsy numbers rose, likely reflecting both detection of small overlooked lesions and closer inspection of ambiguous areas. Since PPV also improved, AI likely prompted more meaningful biopsies rather than unnecessary sampling. The benefit of higher cancer detection clearly outweighs the modest biopsy increase.

Contrary to initial concerns, examination time did not increase after the introduction of AI. Many false‐positive findings were readily recognized as nonneoplastic by endoscopists, which may have limited the need for prolonged detailed inspection. These findings suggest that collaboration between endoscopists and AI can enhance diagnostic performance without prolonging examination time or increasing the burden.

This study has several limitations. As a retrospective design, confounding factors such as equipment differences and varied image‐enhancement use could not be fully controlled. LCI improve early GC detection compared with WLI [24], yet before AI adoption, 20% of procedures used older systems lacking LCI. This study was a retrospective analysis based on routinely collected clinical records. Although AI improves the detection rate of GC case‐level data on endoscopic system usage were not available during the non‐AI period. Therefore, residual confounding due to hardware differences cannot be completely excluded. Even among LCI‐capable devices, enhancement usage varied by endoscopist. These factors may have affected results, though retrospective adjustment was difficult. Second, the study was conducted in Japan, where GC incidence and *H. pylori* infection rates are high. Results may differ in regions with lower GC prevalence, such as Europe or the United States. In such areas, targeted screening of higher‐risk individuals—for example, those with prior *H. pylori* infection—may be necessary. Finally, the AI system provides real‐time support, making it difficult to assess how much it influenced endoscopists' decisions. Future multicenter prospective trials with standardized protocols for endoscopic systems and imaging modes are needed. Furthermore, AI upgrades capable of automatically recording examination time and user responses could facilitate objective evaluation of workflow and decision‐making impact.

In conclusion, AI‐assisted endoscopy significantly enhances the performance of UGI cancer screening, especially for GC. These findings support the integration of AI into population‐based screening programs to promote earlier detection and improved outcomes.

## Author Contributions

**Chihiro Goto:** conceptualization, methodology, data curation, formal analysis, investigation, visualization, writing – original draft. **Ryo Nakagawa:** conceptualization, methodology, supervision, resources, writing – review and editing. **Ryosuke Horio:** data curation. **Akane Kurosugi:** data curation. **Tatsuya Kaneko:** investigation. **Tsubasa Ishikawa:** investigation. **Yuki Ohta:** investigation. **Takashi Taida:** resources, investigation. **Kenichiro Okimoto:** resources, investigation. **Tomoaki Matsumura:** methodology, writing – review and editing. **Jun Kato:** conceptualization, supervision, project administration, writing – review and editing.

## Funding

The authors have nothing to report.

## Ethics Statement

This study was conducted in compliance with the Declaration of Helsinki. The research protocol was reviewed and approved by the ethics committee of Chiba University Graduate School of Medicine, School of Medicine (approval number: M10755). Due to the retrospective nature of the study, the requirement for informed consent was waived by the Ethics Committee. Instead, an opt‐out approach was used for disclosure of patient data. All patient data were anonymized to ensure confidentiality and privacy.

## Conflicts of Interest

Chihiro Goto received honoraria from FUJIFILM Co. Takashi Taida received research grants from AbbVie G.K. Jun Kato received honoraria from Eisai Co. Ltd., Takeda Pharmaceutical Co. Ltd., AbbVie G.K., Zeria Pharmaceutical Co. Ltd., Janssen Pharmaceutical K.K., Mitsubishi Tanabe Pharma Corporation, and JIMRO Co. Ltd., Mochida Pharmaceutical Co. Ltd.

## Supporting information


**Table S1:** Difference in Cancer Detection Rates Between the Non‐AI and AI Groups Stratified by Endoscopist Experience Level.
**Figure S1:**. Flowchart of the Study Population Stratified Into Three Groups According to Endoscopist Experience.

## Data Availability

The data that support the findings of this study are available on request from the corresponding author. The data are not publicly available due to privacy or ethical restrictions.
