# Supplementary material for: Impact of Artificial Intelligence‐Assisted Endoscopy on Screening for Upper Gastrointestinal Cancer in a Large‐Scale Health Screening Facility
Source: Dig Endosc. 2026 May 1;38:e70159. doi: 10.1111/den.70159 (PMC13135021; doi:10.1111/den.70159)
Supplement: Supplementary file 1 — Table S1: Difference in Cancer Detection Rates Between the Non‐AI and AI Groups Stratified by Endoscopist Experience Level. Figure S1:. Flowchart of the Study Population Stratified Into Three Groups According to Endoscopist Experience. [file DEN-38-0-s001.docx]

**Supplementary Table1. Difference in Cancer Detection Rates Between the Non-AI and AI Groups Stratified by Endoscopist Experience Level**

| **Endoscopists experience**  **5-9 years** | **AI group**  **(n=7,645)** | | **Non-AI group**  **(n=11,792)** | | **P value** |
| --- | --- | --- | --- | --- | --- |
| **GC, CDR(%)** | **10** | **0.13%** | **4** | **0.03%** | **0.0150** |
| **GC+EC, CDR(%)** | **14** | **0.18%** | **6** | **0.05%** | **0.0055** |
| **Endoscopists experience**  **10-14 years** | **AI group**  **(n=6,662)** | | **Non-AI group**  **(n=12,441)** | | **P value** |
| **GC, CDR(%)** | **5** | **0.08%** | **6** | **0.05%** | **0.4638** |
| **GC+EC, CDR(%)** | **7** | **0.11%** | **10** | **0.08%** | **0.5817** |
| **Endoscopists experience**  **15 years or more** | **AI group**  **(n=3,395)** | | **Non-AI group**  **(n=8,085)** | | **P value** |
| **GC, CDR(%)** | **3** | **0.09%** | **3** | **0.04%** | **0.2951** |
| **GC+EC, CDR(%)** | **3** | **0.09%** | **4** | **0.05%** | **0.4566** |

GC: gastric cancer, EC: esophageal carcinoma

**Supplementary Figure1. Flowchart of the Study Population Stratified Into Three Groups According to Endoscopist Experience
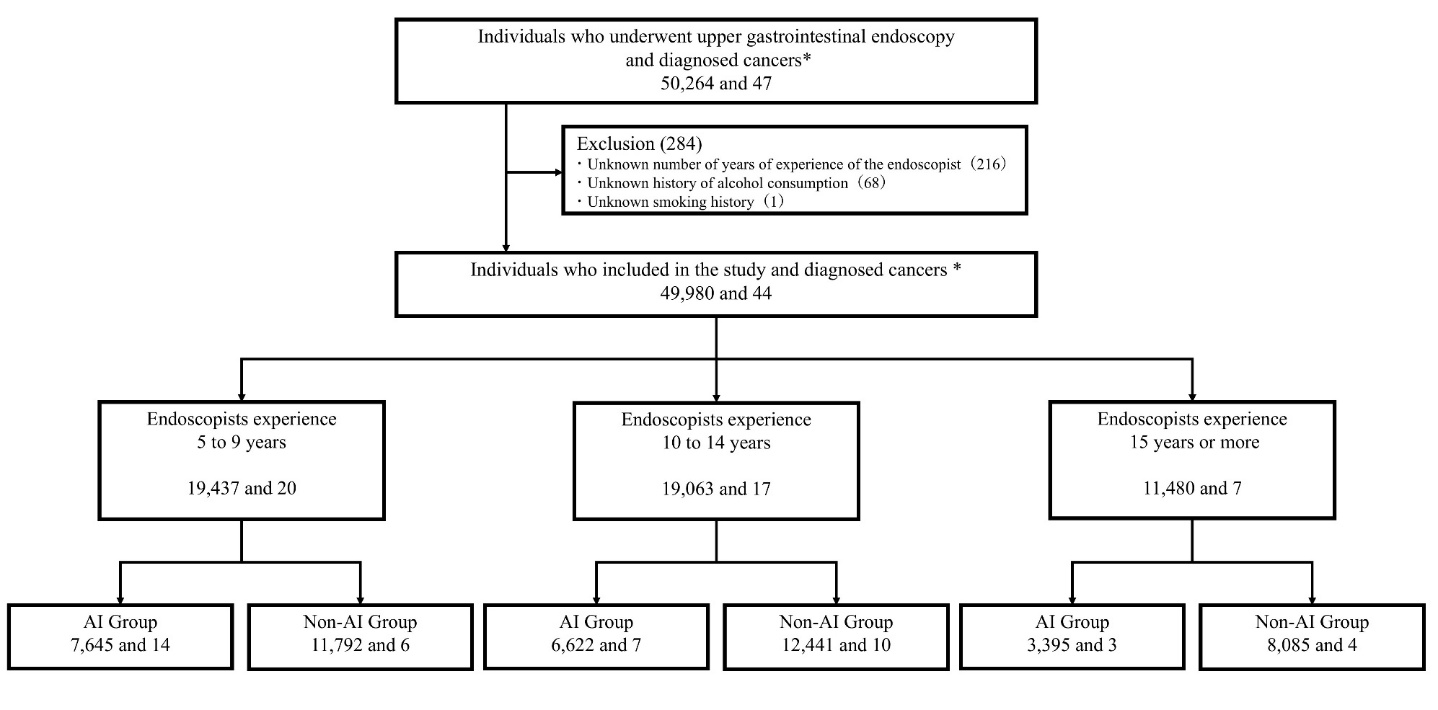
**

*The number of participants diagnosed with gastric cancer or esophageal carcinoma
